# Gain-of-Function and Loss-of-Function Mutations in the RyR2-Expressing Gene Are Responsible for the CPVT1-Related Arrhythmogenic Activities in the Heart

**DOI:** 10.3390/cimb46110767

**Published:** 2024-11-13

**Authors:** Roshan Paudel, Mohsin Saleet Jafri, Aman Ullah

**Affiliations:** 1 School of Systems Biology, George Mason University, Fairfax, VA 22030, USA; 2 School of Computer, Mathematical, and Natural Sciences, Morgan State University, Baltimore, MD 21251, USA; roshan.paudel@morgan.edu; 3 Center for Biomedical Engineering and Technology, University of Maryland School of Medicine, Baltimore, MD 20201, USA

**Keywords:** CPVT1, RyR2, gain-of-function, β-adrenergic, loss-of-function, EAD, alternans, Ca^2+^ dynamics

## Abstract

Mutations in the ryanodine receptor (RyR2) gene have been linked to arrhythmia and possibly sudden cardiac death (SCD) during acute emotional stress, physical activities, or catecholamine perfusion. The most prevalent disorder is catecholaminergic polymorphic ventricular tachycardia (CPVT1). Four primary mechanisms have been proposed to describe CPVT1 with a RyR2 mutation: (a) gain-of-function, (b) destabilization of binding proteins, (c) store-overload-induced Ca^2+^ release (SOICR), and (d) loss of function. The goal of this study was to use computational models to understand these four mechanisms and how they might contribute to arrhythmia. To this end, we have developed a local control stochastic model of a ventricular cardiac myocyte and used it to investigate how the Ca^2+^ dynamics in the mutant RyR2 are responsible for the development of an arrhythmogenic episode under the condition of β-adrenergic (β-AR) stimulation or pauses afterward. Into the model, we have incorporated 20,000 distinct cardiac dyads consisting of stochastically gated L-type Ca^2+^ channels (LCCs) and ryanodine receptors (RyR2s) and the intervening dyadic cleft to analyze the alterations in Ca^2+^ dynamics. Recent experimental findings were incorporated into the model parameters to test these proposed mechanisms and their role in triggering arrhythmias. The model could not find any connection between SOICR and the destabilization of binding proteins as the arrhythmic mechanisms in the mutant myocyte. On the other hand, the model was able to observe loss-of-function and gain-of-function mutations resulting in EADs (Early Afterdepolarizations) and variations in action potential amplitudes and durations as the precursors to generate arrhythmia, respectively. These computational studies demonstrate how GOF and LOF mutations can lead to arrhythmia and cast doubt on the feasibility of SOICR as a mechanism of arrhythmia.

## 1. Introduction

The ryanodine receptors (RyRs) are intracellular Ca^2+^ channels found in the sarcoplasmic or endoplasmic reticulum of cardiac (RyR2) and skeletal muscles (RyR3). They are responsible for releasing intracellular Ca^2+^ for excitation and contraction coupling. Another isoform of RyR, RyR3, is found in brain tissue. RyR isoforms are associated with over 300 mutations and a number of genetic diseases because of those mutations [1,2,3]. Mutations in the RyR2 genes are thought to cause arrhythmia in the heart through alterations to the Ca^2+^ dynamics. CPVT can be caused by an autosomal dominant mutation in the RYR2 gene, and it is designated as type 1 CPVT (CPVT1) [4,5].

### 1.1. Gain-of-Function

The gain-of-function RyR2 mutation accounts for more than 50% of CPVT1 cases [6]. Since the identification of an RyR gene mutation causing CPVT by Priori et al. in 2001, a total of more than 150 different RyR2 mutations have been reported in CPVT patients [7,8,9]. Most of these mutations cluster in three different “hot spots” regions, 176–420 amino acids located in the N-terminal region (domain I), 2100–2500 amino acids of the central region (calstabin2 binding domain II), and after amino acids of 3778–4950 in the C-terminal region (domain III) out of 4967 amino acids [10,11,12,13]. RyR2 as a gene contains 105 exons, and 45 of these exons were reported to have CPVT-causing mutations, and substitution mutations are highest in number [14]. Most of the RyR2 mutations are associated with a gain of function (increase RyR2 opening probability) that leads to the increased release of SR Ca^2+^ into the myoplasm [1,2].

### 1.2. Destabilization of Binding Proteins

Some CPVT1 variants affect the binding between RyR2 subunits altering the opening or closing of the channels. There have been various mechanisms proposed. Yamamoto et al. suggested that the NH2-terminal and the central domains of RyR2 interact as a domain pair and that CPVT-linked RyR2 mutations in either of these domains cause the channels to be hyper-active and hyper-sensitive [15]. Wehrens et al. and Marks suggested that a RyR2-binding protein, calstabin 2 (FKBP12.6), stabilizes RyR2s in wild-type myocytes in which CPVT-linked mutations in FKBP12.6 can disassociate from the RyR2 [16,17].

### 1.3. Store-Overload-Induced Ca^2+^ Release (SOICR)

Jiang et al. hypothesized that in the enhanced-SOICR mutant RyR2, the threshold for store-overload-induced Ca^2+^ release (SOICR) in the SR is lower than in myocytes with WT RyR2 due to the enhanced RyR2 channel sensitivity towards the luminal Ca^2+^ [18]. On the other hand, the sensitivity towards cytosolic Ca^2+^ remains unchanged. The mechanism states that in myocytes with an enhanced SOICR mutant RyR2 with the lower threshold for release, the increase in SR Ca^2+^ load received due to the β-AR stimulation causes the RyR2 channels to open irrespective of the depolarization and spontaneously release SR Ca^2+^ to trigger DADs.

### 1.4. Loss of Function

In contrast, there are some mutations responsible for a loss of function associated with idiopathic ventricular fibrillation [19]. Some studies also suggest that the mutations also increase the sensitivity of the channels to the activating agents (36). Missense mutations, consisting of single-nucleotide substitutions (point mutations) that lead to the substitution of amino acids, are common in RyR2 [20,21,22]. However, in a severe form of CPVT, the deletion of an entire third exon of 35 amino acids also takes place [14,23]. Surprisingly, this deletion does not cause any misfolding or aggregation and is still a gain-of-function mutation [24]. Any mutation that causes the removal of the entire RyR2 is lethal embryonically [25].

The focus of this study is to understand the four proposed mechanisms of CPVT1 caused by a mutation in the gene expressing intracellular Ca^2+^ channels, RyR2s. This study will test the hypotheses that a gain of function that includes increased sensitivity, interdomain unzipping, and an overload threshold change (SOICR) and loss-of-function mutations can both cause arrhythmia through different mechanisms. These variations intensify the function of these channels, increasing their open probability [26,27]. An elevated release of SR Ca^2+^ affects the intracellular Ca^2+^ dynamics and is thought to trigger arrhythmia during exercises or stress. It is believed that an increased SR Ca^2+^ load during rapid pacing combined with the increased RyR2 open probability leads to arrhythmia. However, with the increase in the RyR2 open probability, there is an increase in SR Ca^2+^ leak, which can limit Ca^2+^ accumulation in the SR, complicating this hypothesis. This apparent paradox will be explored using our computational modeling as we address the mechanism of the RyR2 dysfunction approach.

## 2. Materials and Methods

### 2.1. Model Development

Presented here is a whole-cell stochastic model of Guinea pig cardiac ventricular myocyte excitation–contraction coupling that integrates a modified model of stochastic Ca^2+^ dynamics from our published rat and Guinea pig ventricular myocyte models [28,29,30]. A Guinea pig model was developed because it displays a long action potential more similar to human than rat or mouse and it can be experimentally verified. Guinea pigs have some experimental advantages over other long-AP species, such dogs or pigs, relating to housing and cost. This Monte Carlo simulation model uses 20,000 stochastically gating Ca^2+^ release units (CRUs). The CRUs are the cluster of 14 L-type and 49 RyR2 channels coupled with a dyadic subspace. We have integrated RyR adaptation to the gating mechanism of the intracellular Ca^2+^. The ionic current formulations of the new model are adopted from L-R models [31,32,33]. The system 40,002 ordinary differential equations (ODEs) used in this model were solved using the Euler method. The L-type channel was described based on six-state gating mechanisms that incorporate both voltage-dependent activation/deactivation and Ca^2+^-dependent inactivation, similar to Sun et al. [34].

### 2.2. RyR2 Model

**Figure 1:**
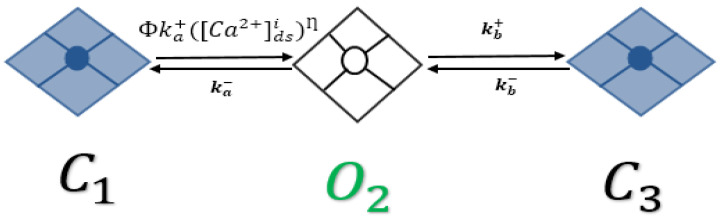
A novel, three-state RyR2 model. C_1_ indicates the closed state. The green O_2_ indicates the open state. C_3_ indicates the adapted state.

The intracellular Ca^2+^ release from the junctional SR is based upon a novel three-state model of the RyR2 that combines RyR adaptation to our previously developed RyR2 model that produced Ca^2+^ sparks (Figure 1). The adaptive state was experimentally proven by Gyroke et al. [35,36]. In a resting AP, the RyRs are in a closed state (C_1_), and when the Ca^2+^ in the dyad increases, the channels are in the open state (O_2_) for a very short period and then go to an adaptive state (C_3_). Upon a further increase in Ca^2+^, the RyRs may return to an open state (O_2_). This RyR2 gating in the model is modulated by cytosolic Ca^2+^ sensitivity and luminal Ca^2+^ dependency.

In the resting phase, all RyR2s stay in the close state (C_1_); with the arrival of Ca^2+^ in the dyadic subspace, the channels activate into the open state (O_2_), and after some time, the channels might inactivate into an adaptive state (C_3_). In this model, the luminal regulation function (Φ) modifies the channel opening rate, SR load, and [Ca^2+^]_SR_ available to be released [37]. RyR2 release flux reaches near its peak with the increase in the pacing frequency [Ca^2+^]_SR_ availability to be released, calculated using the following equation [29]:

(1) $${SR}_{rel}=v_{1}{(P}_{O,RyR})([{Ca}^{2+}{]}_{sr}-[{Ca}^{2+}{]}_{ds})$$

where $v_{1}$ is the maximum Ca^2+^ release via the RyR2 channel, [Ca^2+^]_ds_, the Ca^2+^ concentration in dyadic subspace. Opening the probability of RyR2 ($P_{O,\;RyR}$) is affected by RyR2 adaptation at a higher frequency.

### 2.3. Simulation Protocols

The protocol for this simulation was designed for increased activity of RyR2 due to mutation and increasing sensitivity towards luminal Ca^2+^ sensitivity and dyadic Ca^2+^.

#### 2.3.1. β-Adrenergic Stimulation Protocols

(a) Increased L-type Ca^2+^ influx: The effect of exercise, emotion, and a fight or flight response activates β-adrenergic receptors of protein kinase A (PKA) and increases the L-type channel current [38]. Experiments indicate that the peak L-type current amplitude could increase from 53% [39] or 95% [40] by three-fold [41] when the level of isoproterenol (ISO) increases due to the activation of β-adrenergic receptors in the sarcolemma [41,42]. In adjusting the parameters in our model, a 48% increase in L-type channel permeability (P_dhpr) from the Goldman–Hodgkin–Katz equation was used to simulate the result.

(b) Increase in SERCA2a pump activity: Phospholamban (PLB) inhibits the SERCA2A activities in the SR, but the inhibition is reduced by the stimulation of β-adrenergic receptors, which results in an increased SERCA2A in pump activities [43]. When more Ca^2+^ in the cytosol, due to an increase in Ca^2+^ influx via the L-type current, is present, it is also going to increase the SR Ca^2+^ load [44] with the activation of SERCA2A [45]. To simulate β-adrenergic stimulation, we raised the SERCA rate (A_p_) by 50 percent. The SERCA formulation is given by

(2) $$J_{serca}={2v}_{cycle}A_{p}$$

where $v_{cycle}$ is the cycling rate per molecule, and *Ap* is the concentration of SERCA molecules per liter of cytosol.

#### 2.3.2. Protocols for Mechanisms

After setting up the protocols for β-adrenergic stimulation, we designed the following protocols for four mechanisms: gain-of-function, SOICR, interdomain unzipping, and loss-of-function mutations.

#### 2.3.3. Gain-of-Function Simulation

(a) Increase in P_O_ of RyR2 channels: Mutations in RyR2 increased the opening probability (sensitivity) of the RyR2; in our RyR2 model, we have K^+^ and K^−^ as opening and closing rate constants for RyR2 channels. Potenza et al. [46] recorded a 55% increase in RyR2 phosphorylation during β-AR stimulation. To reproduce the phosphorylation of RyR2, we raised the opening constant (k^+^) by fifty percent in this simulation and applied it in

(3) $$k_{aryr}^{+}=k^{+}\times {\left({\left[{Ca}^{2+}\right]}_{ds}^{\left(i\right)}\right)}^{2.2}\times \left({2.8\times 10}^{-4}{\left[{Ca}^{2+}\right]}_{jsr}^{\left(i\right)}+0.02\right)$$

where i = number of open RyR2 channels (0 to 49), $k_{aryr}^{-}$ = 350, $k_{bryr}^{+}$ = 7.0, $k_{bryr}^{-}$ = 1.0, and k^+^ = 12.

(b) A decrease in the half-maximal point ($K_{m}^{myo})$: The sensitivity of a single RyR2 channel is prompted by local [Ca^2+^]_myo_ and local [Ca^2+^]_sr_. The sensitivity of RyR2 open probability can be represented with a half-maximal point ($K_{m}^{myo})$, a dynamic buffering fraction of the myoplasm as a function of [Ca^2+^]_myo_, also known as the Ca^2+^ dissociation constant from the RyR2. Danielsen et al. adjusted it experimentally by reducing the half-maximal value of [Ca^2+^]_myo_ by 10% [47]. The decrease in half-maximal [Ca^2+^]_myo_ is the main characteristic of a gain of function and varies in different RyR2 mutations. For example, it has been estimated experimentally as 1.5–4-fold less than WT in the N4104K, R4496C, V4653F, and S4153R mutations, and in the RyR2-H2464D mutation, a recorded cytosolic Ca^2+^ sensitivity of RyR2 increased from 19 ± 3% WT to 44 ± 6% in the mutant myocyte [48,49]. These changes were simulated by reducing the value of $k_{aryr}^{-}$ in Equation (3) by 30% (500 − 150 = 350). Apart from these two parameters, the leakiness of RyR2 was adjusted by reducing the value of allosteric coupling ($a_{*})$ by 80% as explained below.

#### 2.3.4. Destabilization of Binding Proteins or Interdomain Unzipping

As per the RyR2-binding protein theory, there is a reduced binding affinity of RyR2 under basal conditions. To reproduce this behavior in our model, we decreased allosteric coupling ($a_{*}$), i.e., the interactions among the homotetramers in the RyR2, by 50% and ~100%. We simulated without a current clamp and checked whether the Ca^2+^ leak was able to generate any DADs during the diastolic phase in both cases. The allosteric coupling factors $X_{oc}$ and $X_{co}$ are given by Williams et al. [28]:

(4) $$X_{oc}=\exp\left\{-a_{*}0.5\left[N_{c}\varepsilon_{cc}-\left(N_{o}-1\right)\varepsilon_{00}\right]\right\}$$

(5) $$X_{co}=\exp\left\{-a_{*}0.5\left[N_{0}\varepsilon_{00}-\left(N_{c}-1\right)\varepsilon_{cc}\right]\right\}$$

where $a_{*}$ is average allosteric connectivity, and $\varepsilon_{cc}$ and $\varepsilon_{oo}$ are the dimensionless free energy of interaction representing free energy experienced by a channel in a closed state C or open state O when it allosterically couples with another channel in the respective states. N_c_ and N_o_ are the number of closed or open states in the CRUs (0 ≤ N_o_ ≤ 49).

The stimulations testing the destabilizing protein mechanism were performed both in resting mode and in current-clamp mode. In resting mode, the initial Ca^2+^ concentration in the SR was increased by two-fold while RyR2’s sensitivity and hyperactivity were added for the current-clamp mode.

#### 2.3.5. Simulation of Store-Overload-Induced Ca^2+^ Release (SOICR)

The CICR is the central phenomenon of the E-C coupling, and it regulates subcellular Ca^2+^ signaling in the myocyte. It is initiated by the RyR2 sensitivity towards [Ca^2+^]_myo_, and luminal Ca^2+^ sensitivity has a major influence on it [50]. To test the SOICR hypothesis that SR Ca^2+^ activates RyR2 channels, the cytosolic Ca^2+^ CICR mechanism was disabled by setting the dyadic Ca^2+^ concentration [Ca^2+^]_ds_ term in the RyR activation equation (Figure 1) to be a fixed parameter (constant) at the resting level by (0.0954 µM). The simulation was run with β-AR stimulation. The SR was loaded with 100% Ca^2+^, and luminal dependence was increased by 90% in the enhanced-SOICR mutant. The goal over here was to slow down the CICR process and to let the SOICR phenomenon get going as claimed by Jiang et al. [18].

#### 2.3.6. Loss-of-Function Mutation

In the lipid bilayer experiment of the loss-of-function mutant, RyR2-A4860G, it was found that RyR2s are very insensitive to the increased luminal Ca^2+^ and the average RyR2 opening probability was below 20% with the Ca^2+^ concentration ranging from 100 nM to 50 mM during β-AR stimulation [19]. Likewise, Zhao et al. [51] also found suppressed P_O, RyR2_ and overloaded SR in the knock-in mouse model with the mutant RyR2-A4860G during ISO treatment. To simulate this change in our model, the luminal dependence of RyR2 was reduced until P_O, RyR2_ went below 20% percent in the β-AR myocyte.

The changes made in various parameters in different mechanisms are listed in Table 1 below. It shows which parameter values were increased, decreased, or stayed the same.

### 2.4. Computational Resources

The PGI CUDA Fortran compiler (www.pgroup.com accessed on 1 July 2023) was used to execute and simulate the program in the Linux platform, Ubuntu operating system. CUDA (compute unified device architecture) is a parallel computing platform and programming language developed for graphic processing units (GPUs) by NVIDIA. The CUDA clusters that we are using in the lab contain Fermi-based C2050 graphics processing cards with CUDA SDK 6.0 and higher. To capture calcium dynamics at a single-channel level, a novel computational algorithm, the Ultra-Fast Markov chain Monte Carlo (UMCMC) method, was used for the stochastic gating from CRUs [52]. The figure plots were made using python (version 3.12, Wilmington, DE, USA) and MATLAB (R2023b, Natick, MA, USA).

## 3. Results

Simulations were performed to compare the different CPVT1 mechanisms (mutant) with the wild-type. While simulating myocytes with the adrenergic receptor activated, the wild-type myocytes showed normal pacing from 1 to 6 Hz. In general, the CPVT myocyte for the various mutants displayed normal behavior up to 5 Hz; however, it developed variations in the AP duration and amplitude as early as 6 Hz. Our explanation of the result below compares plots of AP and its other components between 6 Hz pacing in WT myocytes and mutant myocytes with the different mechanisms with and without adrenergic stimulations in both cases.

### 3.1. Binding Protein Destabilization

**Figure 2:**
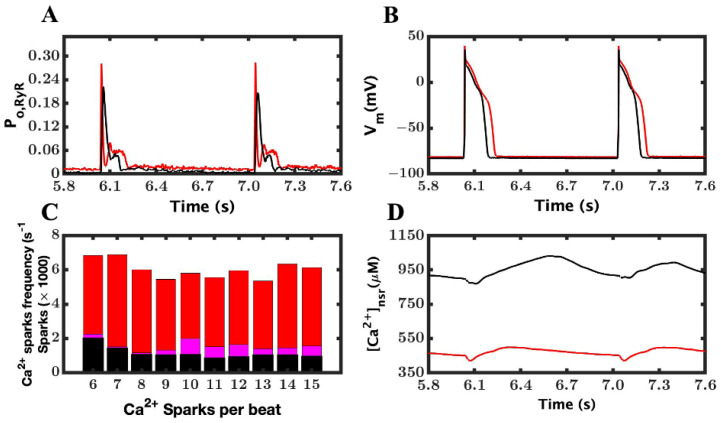
Simulated RyR2 unzipping caused by different levels of allosteric coupling. (**A**) RyR2 open probability. The wild-type is shown in black (100% lowering of AC) and RyR2 unzipping mutant in red. (**B**) Action potentials. With ~100% lowering of allosteric coupling, there is massive spontaneous Ca^2+^ release during the diastolic phase, which can be seen in the RyR2 open probability, but there are no signs of DADs in the AP. (**C**) Number of Ca^2+^ sparks in between 5 and 15 s when AC was lowered by 50% (fuschia) and when AC was lowered by ~100% (red) vs. the wild type (black). In both cases, no DADs were reported during the resting potential with the *p*-value 8.723 × 10^−6^. (**D**) Network SR Ca^2+^ concentration for RyR2 unzipping mutant and wild-type.

The allosteric coupling simulations to produce interdomain unzipping and Ca^2+^ leak were run for 30 s. No appearance of any DADs due to binding protein destabilization of mutant RyR2 was noted. The plots were unable to show any DADs or any other arrhythmic disorders (Figure 2A,B). No change in the membrane potential of the sarcolemma was recorded in the resting potential during the entire simulations. In the ten-second segment from 6–15 s, the Ca^2+^ release both in spark release and non-spark release was recorded. The numbers of Ca^2+^ sparks found during the simulation are plotted in a graph shown in Figure 2C,D. In wild-type simulation (WT), the average number of Ca^2+^ sparks was 129 ± 55.27 and the average Ca^2+^ spark amplitude was 48 ± 0.56 µM. The average numbers of Ca^2+^ sparks were 1575 ± 306 with 50% lower allosteric coupling and 6034 ± 506 when allosteric coupling was further reduced by ~100%. The number of sparks did not display any abnormalities in the resting potential. The average spark amplitudes were 51 ± 0.19 µM and 55 ± 0.85 µM for the 50% and 100% reductions, respectively. The means for these attributes were calculated using Microsoft Excel, and the two-sided student *t*-test was used to compare the means while assuming that there were various variances and sample sizes. The non-spark Ca^2+^ release in both the WT and RyR2 unzipping mutant (regardless of the value of allosteric coupling) was ~19.40 million RyR2 openings from WT and ~19.98 million in the RyR2-unzipping-mutant myocyte, respectively. A ~20% decrease in the SR Ca^2+^ level, as well as smaller peak of the Ca^2+^ transients, in comparison to WT myocytes was also observed.

### 3.2. Loss-of-Function Mutation Generates EADs

**Figure 3:**
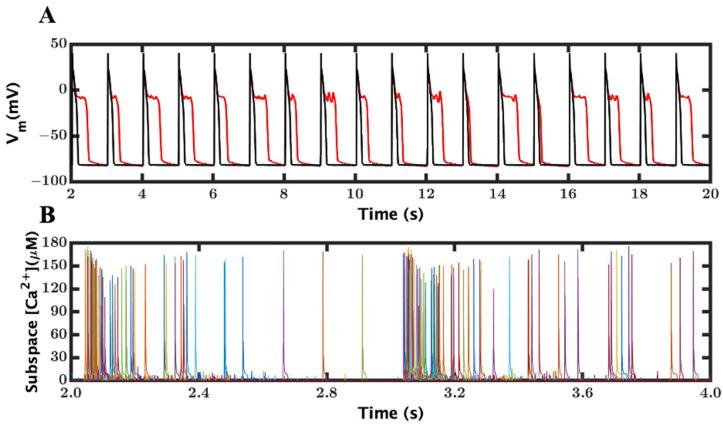
EADs were recorded in a RyR2 loss-of-function mutant myocyte with β-AR stimulation. (**A**) APs with the predominant occurrence of EADs from our model. The wild-type is shown in black and the LOF mutant in red. (**B**) Ca^2+^ sparks for the wild-type. For a comparison of the Ca^2+^ sparks between a normal and EAD AP, please see Alvarez, Jafri, and Ullah Figure 7. In this, the sparks are increased during the EAD as seen is the human ventricular myocyte model [53]. The colors in B correspond to different release units.

The reduction in the luminal Ca^2+^ regulation of RyR2, to simulate a loss-of-function (LOF) mutation, resulted in SR Ca^2+^ release of a longer duration and with slower fall due to a low open probability of RyR2, which resulted in a longer APD (202.18 ± 3.6 ms in LOF mutant vs. 189 ± 1.3 ms WT). The average spark duration increased (32.84 ms LOF mutant vs. 19.31 ms WT) in the 1 Hz simulation even before β-adrenergic stimulation. For mutant myocytes, the luminal Ca^2+^ dependency was lowered (~70%) to have the RyR2 opening rate of ~0.20 in the simulation based upon Jiang et al. [19]. With a β-adrenergic lower RyR2 open probability and increased L-type influx, it resulted in increased APD and spark durations (Figure 3) (APD, 202.18 ± 3.6 ms and spark duration, 48.25 ms), and it was repolarizing and depolarizing to develop an EAD within a beat. This showed that the recorded average APD was huge in comparison to the WT, as well as the LOF mutant myocyte before β-AR stimulation.

**Figure 4:**
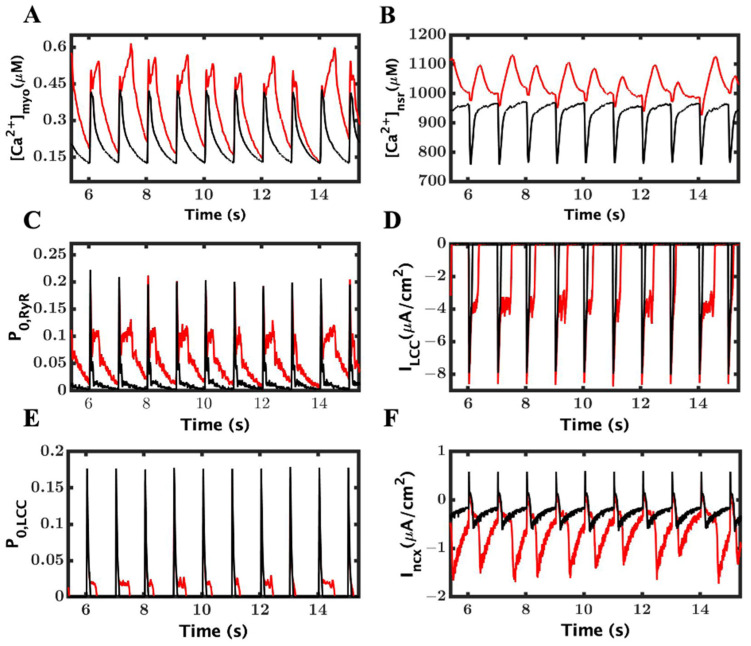
Prolonged and larger-amplitude Ca^2+^ transients and altered time courses of Ca^2+^ handling model components during EADs. (**A**) An inconsistent-looking peak myoplasmic Ca^2+^ transient. The wild-type is shown in black and LOF mutant in red. (**B**) Abnormal but relatively loaded NSR. (**C**) A longer opening of RyR2 receptors. (**D**) A large and longer L-type current (**E**). A longer opening of L-type channels shows activation and reactivation in the same beat. (**F**) An elongated electronegative current I_ncx_.

The peaks of the myoplasmic Ca^2+^ transients were higher in the LOF mutant myocyte compared to the WT, and this increase was exaggerated in the extended APs due to the prolonged Ca^2+^ release from RyR2 channels (Figure 4A,C). The peak diastolic SR [Ca^2+^] was elevated (Figure 4B). Also, with the longer APs, the duration of L-type Ca^2+^ increases, which is followed by a greater elevation in the peak diastolic SR [Ca^2+^] (Figure 4E). With the prolonged APs and L-type Ca^2+^ current, there is also a prolonged and increased inward current due to I_ncx_ (Figure 4F).

### 3.3. Ionic Mechanism of EADs

**Figure 5:**
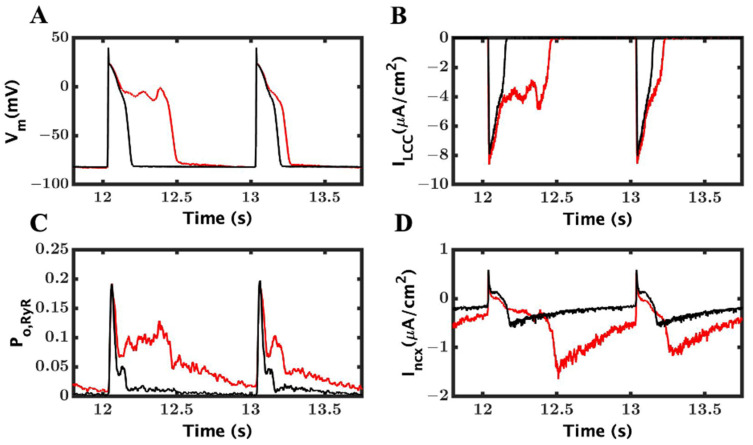
Late reactivation of LCC and SR Ca^2+^ release is essential to generate EADs. (**A**) AP with EADs. The wild-type is shown in black and LOF mutant in red. (**B**) L-type current with late reactivation. (**C**) RyR2 channels activated by LCC current and spontaneous Ca^2+^ release during diastole. (**D**) Loss-of-function mutation is causing an elongated I_ncx_ current due to late release of SR Ca^2+^.

There are thee Ca^2+^-related mechanisms to generate EADs explained in the literature: reactivation and reverse repolarization of L-type current, spontaneous SR Ca^2+^ release, and predominantly inward I_ncx_ current [54]. In our model, the EADs (Figure 5A) were produced in conjunction with reactivation of the L-type current (Figure 5B). At approximately the same, the RyR2 channels were reactivated (Figure 5C) along with an increased I_ncx_ current (Figure 5D). In fact, I_ncx_ is supported by almost double amplitude in EADs over the WT myocyte (209 ± 0.18 vs. 1.10 ± 0.05).

To determine which ionic component was responsible for triggering EAD with the loss-of-function mutation, we observed the time of initiation of AP, I_LCC_, I_ncx_, and RyR2 P_O_ by zooming Figure 3 for ten different EADs and recorded them into a table (Table 2) below. For an example, in the first EAD on the left (EAD1—Figure 5A), it was observed that the L-type current began at 12.118 s and it was followed by RyR2; more channels were open at 12.1359 s, and then, I_ncx_ was further activated by the cytosol Ca^2+^ and depolarized at 12.1445 s, and ultimately, AP developed EAD at 12.1508 s. The other EADs followed a similar trend to trigger EADs like the first one. The difference in the RyR2-I_LCC_ and the I_ncx_-RyR2 initiation times showed a consistent delay across the EADs. The data clearly suggests that the late reactivation of L-type channels triggered RyR opening and the EADs. The difference between RyR2 Po with I_LCC_ (mean 0.021 ± 0.008) and I_ncx_ with RyR2 P_o_ consistent which also support our claim of late reactivation of I_LCC_ and release of SR Ca^2+^ are responsible for generating EADs.

**Figure 6:**
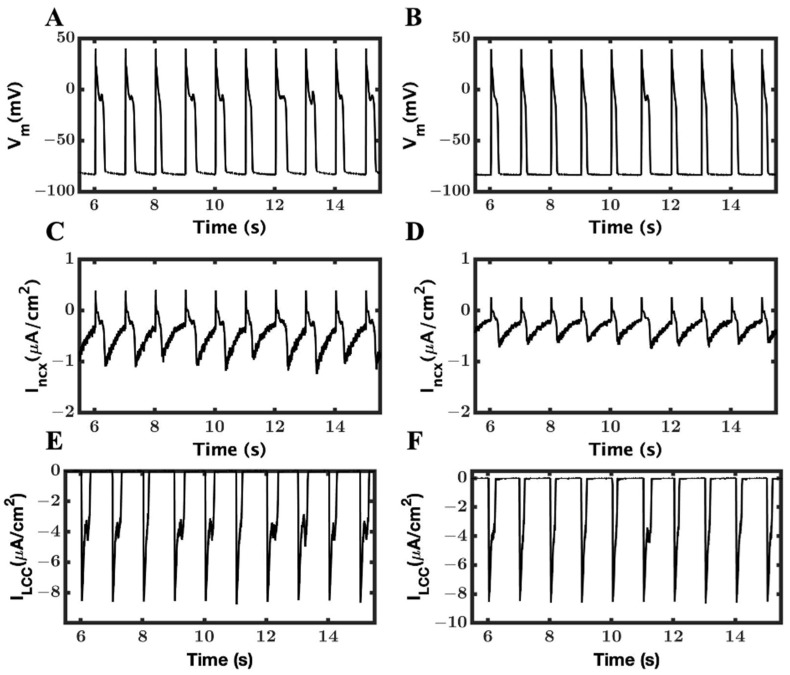
The role of I_ncx_ in the reduction of EADs. 25% and 50% depletions in I_ncx_ currents were carried out to find out whether blocking of I_ncx_ has any role in reducing or preventing formation of EADs. (**A**) Membrane potential (V_m_) with 25% blocking of the I_ncx_. (**B**) Membrane potential (V_m_) with 50% blocking of the I_ncx_. (**C**) Na^+^–Ca^2+^ exchanger current (I_ncx_) with 25% blocking of the I_ncx_. (**D**) Na^+^–Ca^2+^ exchanger current (I_ncx_) with 50% blocking of the I_ncx_. (**E**) L-type Ca^2+^ current (I_LCC_) with 25% blocking of the I_ncx_. (**F**) L-type Ca^2+^ current (I_LCC_) with 50% blocking of the I_ncx_.3.4. Role of I_ncx_ in the minimization of EADs.

To understand the role of the ionic mechanism of I_ncx_ to generate EAD in the above simulations (Figure 3), we blocked the I_ncx_ current by 25% and 50% in a new set of simulations. When I_ncx_ was reduced by 25%, we could shrink APD (338.97 ± 54.5 ms from 438.97 ± 123.8 ms), but the APs (Figure 6A) still had many EADs remaining. After a 50% reduction in I_ncx_, the APD (Figure 6B) was further lowered (238.7 ± 38.55), and many EADs were significantly disappeared from the APs. The amplitude of I_ncx_ (Figure 6C,D) also dropped to 1.50 ± 0.08 from 2.05 ± 0.18 at 50% and 25%, respectively. The duration of the L-type and, similarly, the amplitude of the I_ncx_ current were also heavily changed, going to 50% from 25% (Figure 6C,D), with 25% less I_ncx_ and reached 0.95 ± 0.04 with 50% less I_ncx_. The reduction in the I_ncx_ also affected the duration of L-type channels (Figure 6E,F); it was 314.75 ± 109.20 ms to 272.55 ± 57.19 ms to 169.09 ± 30.22 ms in the original, 25%, and 50% reduced I_ncx_, respectively.

**Figure 7:**
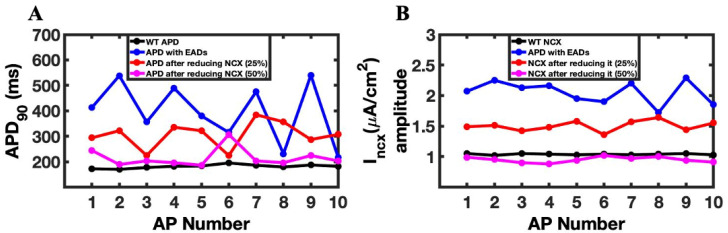
Blocking of I_ncx_ current by 25% and 50% reduced the frequency of EADs occurring in RyR2 loss-of-function mutation. (**A**) APD_90_ in WT myocyte (black), EADs with β-AR stimulation, and original I_ncx_ (blue), APD_90_ after reducing I_ncx_ by 25% (red), and 50% (fuchsia), in both the reduction β-AR stimulation, was unchanged. (**B**) I_ncx_ amplitude in WT (black), with original NCX value during EADs (blue), after reducing it by 25% (red) and by 50% (fuchsia).

A comparison of APD90 (Figure 7A) before and after reducing the I_ncx_ provides a clearer clue of how I_ncx_ plays the role in removing the heavy occurrence of EADs from the AP. The amplitude of I_ncx_ was greatly reduced (Figure 7B), but it was able to successfully eliminate most of the EADs.

The reduction in the I_ncx_ current reduced the frequency of EAD occurrence. The reduction of APD, activation time of LCC, and the spark duration were also decreased with a lower value of I_ncx_. We recorded a significant difference in the [Ca^2+^]_myo_ peak transient from a normal I_ncx_ to 25% reduction but when comparing a 25% to 50% reduction (66.7 ± 2.41 (EAD), 63.6 ± 2.1 (50%), and 63.5 ± 1.8 (25%)), it was less significant in compared to the EAD reduction. Similar tendencies were found for the opening probabilities of LCC and RyR2, SR Ca^2+^ level, and even in the I_Na_ and inactivation gate.

### 3.4. SOICR Mechanism Is Unable to Develop Any Arrhythmia

**Figure 8:**
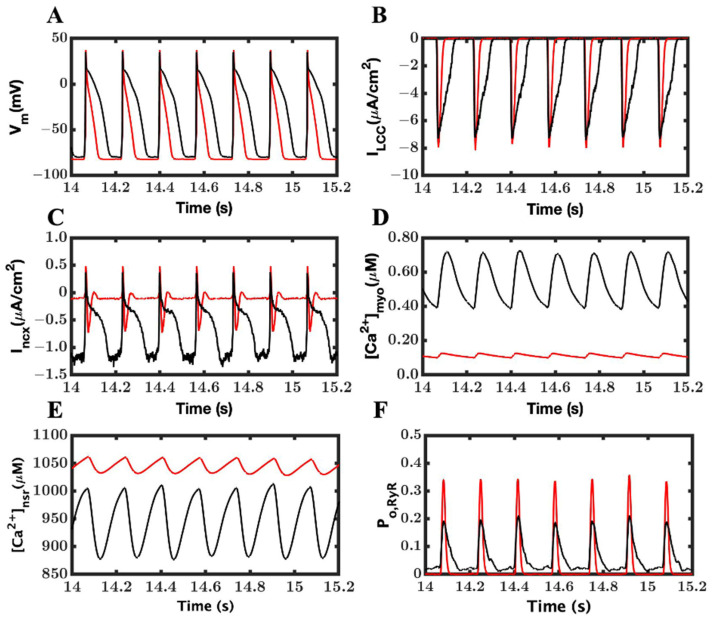
Store-overload-induced Ca^2+^ release (SOICR) simulation: the model was unable to predict that the SOICR mechanism is responsible for any CPVT1 in the RyR2 mutation. (**A**) Membrane potential (V_m_). The wild-type is shown in black and SOICR mutant in red. (**B**) Na^+^–Ca^2+^-exchanger current (I_ncx_). (**C**) L-type Ca^2+^ current (I_LCC_). (**D**) Myoplasmic Ca^2+^ concentration ([Ca^2+^]_myo_). (**E**) RyR2 open probability (P_O, RyR_). (**F**) Network SR Ca^2+^ concentration ([Ca^2+^]_nsr_).

The next set of simulations tested whether changes in the SR [Ca^2+^] can trigger release through luminal Ca^2+^ sensitivity when the RyR2s were not activated by cytosolic Ca^2+^. The main characteristic of this mechanism is that SR Ca^2+^ overload causes spontaneous Ca^2+^ release and spills over to the cytoplasm. In this simulation, we have tested the SOICR mutant myocyte by keeping the dyadic subspace Ca^2+^ at a resting level to keep the CICR mechanism inactivated. The simulation produced an action potential without a plateau (Figure 8A) (APD = 74.92 ± 1.32 ms) AP; the AP peak (38.07 ± 0.02 mV) was a little higher for 6 Hz pacing. The voltage-gated L-type channel, which was alone responsible for an AP, was activated fully (Figure 8B) here. Generally, AP gets shorter with higher pacing because the release of SR Ca^2+^ provides a negative feedback mechanism but it does not happen if there is no CICR. The Na^+^–Ca^2+^ exchange current (Figure 8C) depends upon cytosolic Ca^2+^, but there was no plateau phase because of the absence of SR Ca^2+^ release, which made it shorter and quicker. The cytosolic Ca^2+^ level was highly affected due to the SOICR mechanism; in higher pacing, it is supposed to have a higher concentration of [Ca^2+^] (Figure 8D), but the variation in each beat was tiny. In the simulation, we also found greatly reduced RyR2 channel opening (Figure 8E), and there was a small variation in the SR Ca^2+^ (Figure 8F). But we counted the Ca^2+^ sparks in each beat, and the average number was 1176 ± 48. When CICR was inactivated, cytosolic Ca^2+^ was not enough for the coordinated openings of mass RyR2 due to many SR Ca^2+^ releases that could not yield the sparks. Even with the very low numbers of sparks, their average per beat amplitude was short (55.72 ± 1.02 vs. 60.55 ± 1.56, WT).

### 3.5. Increased RyR2 Opening Probability Due to Gain-of-Function Mutation Is Accompanied by Altered Excitation–Contraction Coupling Variability

**Figure 9:**
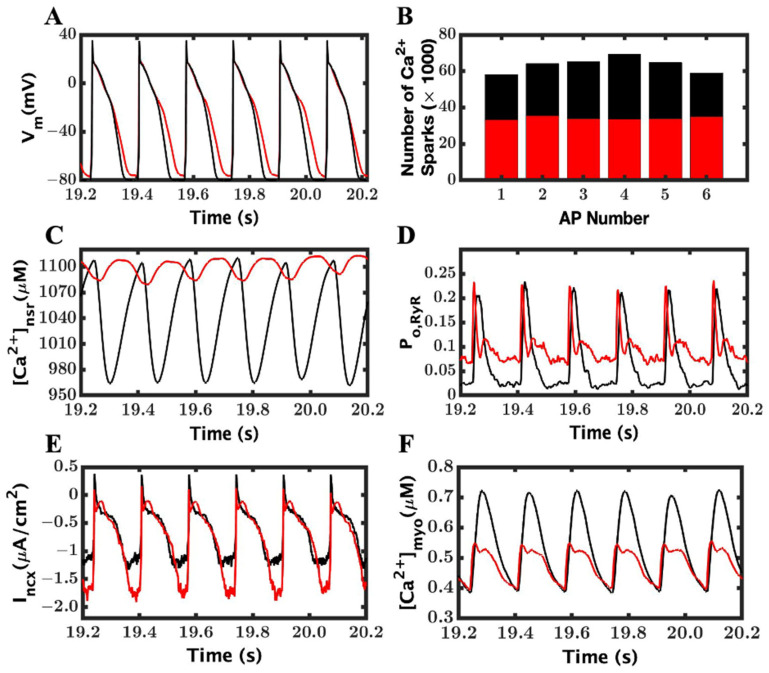
Intracellular Ca^2+^ dynamics are greatly disturbed due to β-adrenergic stimulation in the gain-of-function mutant myocyte described in Table 1. (**A**) AP clearly shows variations in the beats; the beats displayed variations in amplitude and duration in APs. The wild-type is shown in black and GOF mutant in red. (**B**) The Ca^2+^ spark count varies between beats with the beat number corresponding to the beats shown in the other panels. (**C**) NSR Ca^2+^ level ([Ca^2+^]_nsr_), (**D**) opening probability of RyR2 channels, and (**E**) Na^+^–Ca^2+^ exchange current (I_ncx_). (**F**) Cytoplasmic Ca^2+^ level ([Ca^2+^]_myo_).

Simulations to test the effects in the gain-of-function (GOF) mutant myocyte caused by gain-of-function mutations, such as interdomain unzipping, under β-adrenergic stimulation were carried out at pacing rates between 1 Hz and 6 Hz for both WT and GOF mutant myocytes. The wild-type myocytes paced in both the control and β-adrenergic receptor stimulation showed normal results except frequency-based changes, such as a decrease in APD, an increase in I_ncx_, and intracellular Ca^2+^ level increase. The control pacing of the GOF mutant myocyte obtained similar results to the wild-type myocytes. The β-adrenergic stimulation in the GOF mutant myocyte showed a steady train at a lower pacing rate. When the pacing increased to a 6 Hz frequency, the variations were observed in the APs of other ionic currents and Ca^2+^ transients (Figure 9). There were also transient periods of alternans.

The variation in APs were represented in the form of different AP peaks with longer and shorter APDs, respectively (Figure 9A). The number of Ca^2+^ sparks also vary between beats with the higher number of sparks corresponding to the larger amplitude beats (Figure 9B). The β-AR increased the Ca^2+^ influx to the myocyte, but the availability of SR Ca^2+^ and higher SR emptying are seen during the larger APs that showed the larger number of sparks (Figure 9C). In the simulations, we found that the average spark duration is very long in the GOF mutant, 328 ms compared to 19 ms in the WT, and there was significant Ca^2+^ spark-based leak in the diastolic phase, as seen by the increased open RyR open probability (Figure 9D) compared to the normal myocyte. The Na^+^–Ca^2+^ exchanger current (I_ncx_) in the GOF mutant shows increased Ca^2+^ extrusion late in the AP, generating more depolarizing inward current than the wild-type (Figure 9E), and myoplasmic Ca^2+^ transients (Figure 9F) were also affected with the alternation in AP.

**Figure 10:**
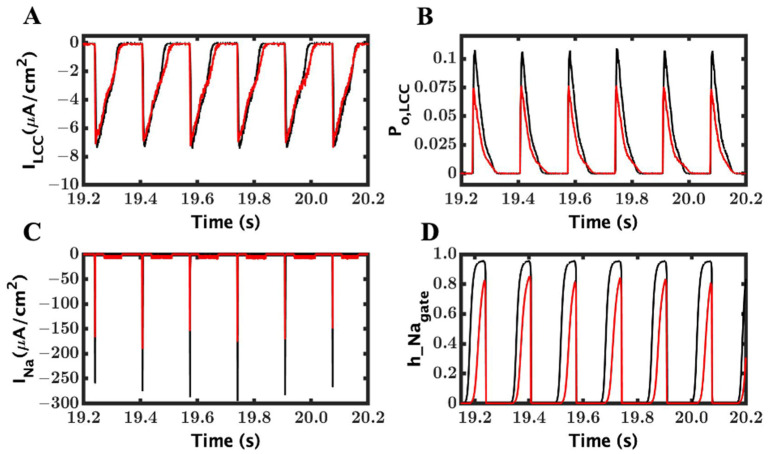
The opening probability of LCC did not control the amplitude of the L-type current and increased cytosolic Ca^2+^ raised the activity of the Na^+^–Ca^2+^ exchange current. (**A**) L-type current. The wild-type is shown in black and GOF mutant in red. (**B**) L-type channel open probability (P_O, LCC_). (**C**) Na^+^ current, I_Na_. (**D**) Na^+^ channel inactivation gate, h_Na_gate_.

The simulations also showed beat-to-beat variations in the L-type Ca^2+^ current and increased late L-type current in the GOF mutant compared to the wild-type (Figure 10A). There is a decrease in the L-type peak open probability and increased late L-type channel open probability (Figure 10B). The larger L-type Ca^2+^ open probability (Figure 10B) occurs when the Na^+^ current amplitude is larger, which corresponds to the wild-type (Figure 10C). After a longer AP, as seen in the second beat in Figure 9A, there is less recovery from inactivation (Figure 10D) and hence less I_Na_ current (Figure 10C).

**Figure 11:**
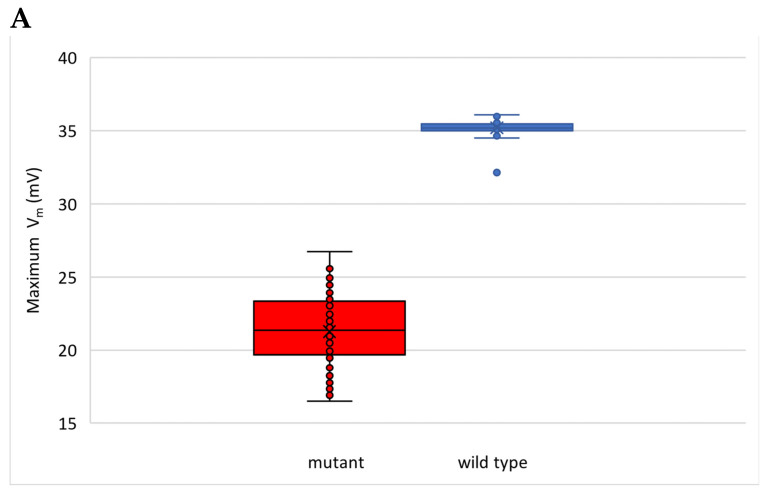

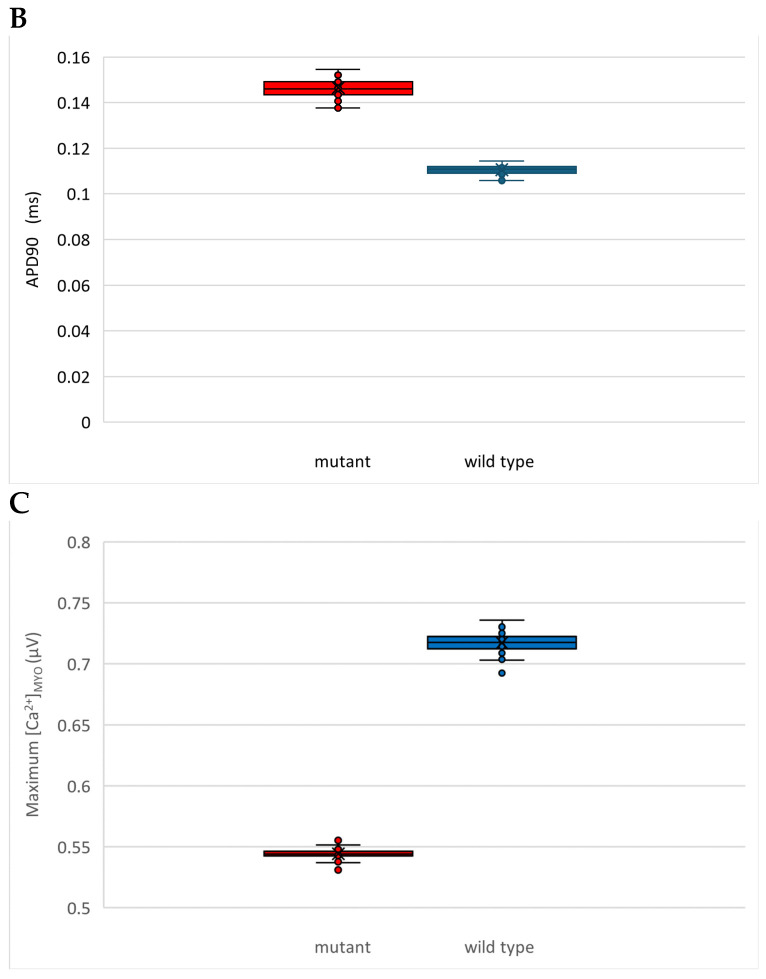
Variations: (**A**) AP variability and (**B**) AP duration at 90% recovery (APD90). (**C**) Ca^2+^ transient amplitude variability. The full time series for the simulation used to generate Figure 11 are found in Appendix A Figure A1 and Figure A2 (beat 80–180).

To determine whether the observed variations in action potentials (APs) were indicative of alternans or not, we conducted several statistical analyses. As depicted in Figure 11A, our findings revealed a decrease in AP peak and an increase in variability of the AP peak among the GOF mutant group compared to the wild-type (WT) group. Figure 11B demonstrates that the APD90 of the GOF mutant shows prolongation of the action potential and increased variability. On the other hand, Figure 11B indicated a decreased Ca^2+^ amplitude and decreased variability in Ca^2+^ transient amplitude in the mutant compared to the wild-type.

## 4. Discussion

This computational modeling study explored the role of gain-of-function and loss-of-function mutations in the RyR2 on excitation–contraction coupling. This is the first comparison of the competing mechanisms tested through computational modeling. Many of the known mutations in RyR2 increase the opening probability of RyR2 as it is activated by cytosolic Ca^2+^. The activation of β-AR brings an increase in the pacing rate of the heart and increases the Ca^2+^ flux per unit time. More Ca^2+^ in the cytosol increases the rate of the SERCA pump, and it replenishes extra Ca^2+^ back to the SR. The pairing of high-SR Ca^2+^ load with hyperactive RyR2 produces large Ca^2+^ transients, which affect Ca^2+^ dynamics in the myocyte. This condition contributes to the development of variations, and it triggers CPVT1 in the heart. In assessing four different hypotheses to explain the mechanisms of the RyR2 mutation and CPVT1, we have seen that the modulation of the RyR2 open probability is greatly influenced by the RyR2 mutation affecting its response towards the cytosolic Ca^2+^ sensitivity and luminal Ca^2+^ dependency. There should be primarily two categories of RYR2 mutations to answer all the questions on RyR2 mutations and CPVT1: gain-of-function and loss-of-function. The destabilization of the binding protein mutation is a part of a gain-of-function mutation, and the diastolic leak due to this mutation is responsible for the low level of SR Ca^2+^ but not cardiac instabilities. The literature illustrates that much more work has been performed on gain-of-function mutations, and still many researchers consider that the LOF mutation does not exist. We have tested the LOF mechanism in our model and retrieved those experimental findings that are plausible; more experiments would shed light on it. There are still many unanswered questions on arrhythmia sudden cardiac death; the model displayed that the gain-of-function mutation was responsible for the CPVT1 in the mutant myocyte by developing increased variation in the AP. The LOF mutation also triggers arrhythmia by developing EADs. The interdomain unzipping or binding protein destabilization due to a mutation in channel binding proteins is part of the gain of function but deals with the RyR2 channels during the diastolic phase. We did find an increase in the Ca^2+^ leak due to destabilization of the closing of channels during diastole, but it could not have enough leak to depolarize the membrane to trigger DADs and arrhythmia. We did not find this to believe in the SOICR mechanism; we agree with most of the scientific community that CICR is the sole mechanism of excitation–contraction coupling in the cardiac myocytes. An increase in SR luminal Ca^2+^ sensitivity can increase RyR openings, but CICR is needed to allow this to become a Ca^2+^ transient. Researchers have proposed two main mechanistic themes to explain how the dysfunction of mutant RyR2 causes CPVT: (1) gain of function, which includes interdomain unzipping and overload threshold change (SOICR), and (2) loss of function.

### 4.1. Destabilizations of Binding Proteins and Interdomain Unzipping

Some CPVT1 variants affect the binding between RyR2 subunits altering the opening or closing of the channels. Yamamoto et al. reported that the NH2-terminal (N: 0–600) and the central domains (C: 2000–2500) of RyR2 interact as a domain pair and either of these domains can have CPVT-linked RyR2 mutations, which modify the channels to be hyper-active and hyper-sensitive [15]. They were able to synthesize a peptide, DPc10, having an RyR2 mutation extending from C: 2460 to C: 2495 (Gly^2460^ to Pro^2495^) in a rabbit sequence. The DPc10 is one example of the mutation that causes the unzipping of RyR2 and destabilization of the channel. This defect unfastens the zipping in RyR2 channels required for RyR2 closure during the diastolic phase resulting in an increased Ca^2+^ leak from the SR, which ultimately causes the development of DADs (Delayed Afterdepolarizations) [55,56,57]. The mutant myocyte in the systolic phase starts with low SR Ca^2+^_,_ and there is a significant increase in the sensitivity of the RyR2 and longer duration of Ca^2+^ release [58].

Similarly, Wehrens et al. proposed that a RyR2-binding protein, calstabin 2 (FKBP12.6—C: 2331–2438), stabilizes RyR2s in wild-type myocytes [17]. It is believed that FKBP12.6 maintain a closed state (resting phase) by tightly binding the RyR2 domains, but the binding affinity of the FKBP12.6 protein is reduced in mutant RyR2s. Each of four FKBP12.6 molecules can bind to one tetramer of RyR2 [16]. There are two types of proposed mechanisms of FKBP12.6 destabilization. It is understood that the PKA-induced phosphorylation of RyR2 leads to the dissociation of the FKBP12.6 protein, which increases the open probability of RyR2 channels by increasing its sensitivity towards Ca^2+^ activation [59]. The reduced RyR2-binding affinity of FKBP12.6 causes abnormal leaks of Ca^2+^ during the diastolic phase, developing DADs with β-adrenergic stimulation [18,60,61]. The second mechanism involves reduced binding of the DPc10 or FKBP12.6 proteins to the RyR2, each affecting the binding of the other protein [62]. A mutation in the binding sites is termed a gain-of-function mutation due to the hyperactivity and premature release of SR Ca^2+^ via RyR2 [23].

From our simulation results, we found that the binding protein mutations were unable to produce any arrhythmogenic activities in the simulations. George et al. [58], with their study of three CPVT-related RyR2 mutations, reported that the RyR2/FKBP12.6 interaction was undamaged due to the mutations and acted like WT myocytes. They did not find any abnormality in SR Ca^2+^, [Ca^2+^]_SR_, or cytosolic Ca^2+^, [Ca^2+^]_myo_ in those mutant myocytes during the resting phase. We were also unable to distinguish any changes both in WT and mutant myocytes with the comparison of AP or another ionic current because there were no abnormal activities with the leak. But with the analysis of Ca^2+^ sparks, we found a higher number of Ca^2+^ sparks during diastole in the mutant myocyte than in the WT one, but those sparks were not enough to trigger DADs.

### 4.2. Store-Overload-Induced Ca^2+^ Release (SOICR)

The store-overload-induced Ca^2+^ release (SOICR) hypothesizes that enhanced RyR2 channel sensitivity towards the luminal Ca^2+^ can trigger SR Ca^2+^ release [18]. In this mechanism, the sensitivity towards the cytosolic Ca^2+^ remains unchanged. From our simulation results, we found that the gain-of-function mutation to cause changes in beat-to-beat variability and the SOICR mechanism failed to activate RyR2 receptors by increased sensitivity in low or null CICR. Based upon our findings we could not support the SOICR as a mechanism of CPVT1 mutation in the genes expressing the RyR2 protein. The SR sequestered Ca^2+^ to a high enough level to activate spontaneous Ca^2+^ release and to cause DADs. This is because the increased RyR open probability found in gain-of-function RyR2 mutants leads to increased Ca^2+^ leak from the SR in the form of Ca^2+^ spark and non-spark RyR2 opening as demonstrated in previous work [28].

### 4.3. Gain-of-Function Mutants

The Ca^2+^ spark analysis helped us to understand every basis of this mechanism at the subcellular level and to figure out a variation in the spark’s numbers and amplitudes in beat to beat or second to second. The gain-of-function mutation in the RyR2 proteins that increases the opening probability of RyR2 channels and increase propensity towards the cytosolic Ca^2+^ ends up developing an arrhythmia during rapid pacing with β-AR stimulation. From our model, we found that the gain-of-function mutation causes cardiac variabilities as an indication of those arrhythmias. Similarly, the loss-of-function mutation, which lowers the luminal dependency of RyR2 channels, could cause EADs as the signals of arrhythmia. It was also observed the reducing the value of I_ncx_ in the AP with EADs minimizes the frequency of their occurrence working in synergy with I_LCC_.

Increased RyR2 phosphorylation produced increased AP peak variability and transient periods of alternans (alternate weak/strong beats). D. M. Bers [63] explained SR Ca^2+^ load as the Ca^2+^ release regulation factor in excitation–contraction coupling and CICR. It fully contributes to the underlying mechanism of arrhythmogenic disorders. Diaz et al. [64] also reported that a variation in the SR Ca^2+^ content is enough to produce cardiac alternans.

The combined effect of the mutation in RyR2 and catecholaminergic stimulation is the triggering of an arrhythmia. The mutations cause a premature or prolonged release of SR Ca^2+^ in the cytosol. At the molecular-level study of the number of CPVT1 mutations, the majority of them are the gain-of-function mutations [2,24]. It is necessary to notice that CPVT1 occurs during rapid pacing of the heart under the influence of adrenergic stimulation, and it has to do with the SR Ca^2+^ load. During CPVT, the myocytes are pacing at a rapid rate and a fraction of DI shortens, which allows less time for replenishing SR, the removal of Ca^2+^, and complete relaxation of the ventricles. The rapid pacing elevates the diastolic SR Ca^2+^ level [65,66]. In previous work, during rapid pacing, we have found that there is an increase in the SR Ca^2+^ load in higher pacing alone but with adrenergic stimulation plus rapid pacing, there must be extra Ca^2+^ in the SR [67,68]. But the simulation showed that there was no unexpected increase in the diastolic Ca^2+^ level, which means that there should not be a Ca^2+^-overload-related leak. Danielson et al. [47] reported that the SR Ca^2+^ content is responsible for the leak of Ca^2+^ to generate DADs but found no increased SR Ca^2+^ content in the RyR2 mutant heart during both rapid pacing and adrenergic stimulation, opposite to the WT myocyte. This supports the results we obtained from our model. Williams et al. also reported that the SR Ca^2+^ level depends upon the RyR2 open probability (Po); higher Po lowers the SR Ca^2+^ quantity. Our simulation also could not find an increased SR Ca^2+^ level in the mutant myocyte compared to the wild-type one. Bigger Ca^2+^ sparks are generated in mutant myocytes than wild-type myocytes in each beat. When mutant myocytes paced rapidly during adrenergic stimulation, we recorded an alternate availability of SR Ca^2+^ in successive beats, which resulted in both amplitude and APD variability. However, no variations were recorded during the slow pacing. This occurs because with the increased pacing rate, there is less time for Na^+^ channels and L-type Ca^2+^ channels to recover from inactivation accompanied by an increased diastolic cytoplasmic Ca^2+^. This increases the number of late and diastolic Ca^2+^ sparks increasing the stochasticity in the model. The stochasticity in the Ca^2+^ activates I_NCX_ in a more variable way resulting in more variability in L-type Ca^2+^ activation. The end result is the changes in variability observed. There are well-recognized clinical observations that electrical instability in a short cycle length (higher beating rate) is more likely to deteriorate into ventricular fibrillation (VF) [69], and almost all VFs are preceded by ventricular tachycardia (VT) [70,71]. The results from our model show that variabilities in rapid pacing bring CPVT and it may end in VF or SCD.

### 4.4. RyR2 Loss-of-Function Mutation

Gomez and Richards proposed the loss-of-function hypothesis based upon “non-conventional” findings of Thomas et al. Their hypothesis states that the AP elongation occurs because of the combination of the normal peak and prolonged Ca^2+^ release in different myocytes [72,73]. The ventricular arrhythmia with the RyR2 mutation can also occur with the loss-of-function mutation, which causes a decrease in the Ca^2+^ release during systole resulting in a gradual overload of Ca^2+^ in the SR [19,51,74]. After a few beats, the extra Ca^2+^ in the SR randomly releases a burst of Ca^2+^, which creates EADs to trigger arrhythmia.

From our simulation results, we found that the loss-of-function mutation causes EADs. It is believed that the prolonged Ca^2+^ release translated into EADs, but it is required to unload SR Ca^2+^ and prevent SR overfilling [51]. Similarly, a longer L-type current (Figure 5D) brings an excess influx of extracellular Ca^2+^, which elongates the plateau phase in the AP with the β-AR stimulation of L-type channels. The opening probability of L-type channels (Figure 5E) shows activation and reactivation in the longer beats. Another highly important component that contributes to elongated APD is the Na^+^–Ca^2+^ exchange current, I_ncx_ (Figure 5F). Because of the electrogenic nature of I_ncx_, it brings an extra positive charge in the myoplasm to remove excess Ca^2+^ from the cytosol. It is not always true that the lengthening APD causes EADs [75], but in our model, we saw that the elongated APD increased the chances of reactivation of L-type channels (Figure 5D). It has been found that the EAD oscillations vary with the time and the last oscillation is always larger than preceding oscillations and we have that in our result. As mentioned by Weiss et al., I_ncx_ works in synergy with I_LCC_. However, the dynamics are more complex. We believe the opposite is also true, that the I_LCC_ entertains a positive feedback mechanism from the I_ncx_ [54]. Zhao et al. also found that when treating myocytes with an I_ncx_-inhibiting drug, the APD was decayed by 75–90 percent and EAD incidents were decreased drastically [51].

In the case of the alternans, Chudin et al. [76] described that in rapid pacing, increased intracellular Ca^2+^ accelerates the inactivation of L-type channels. In the simulation, the LCCs were inactivated when higher-load SR Ca^2+^ was released alternately and more Ca^2+^ in the cytosol played the role to inactivate them. When there is enough SR Ca^2+^ available for the CICR mechanism, a strong beat will be produced and less SR Ca^2+^ will be available for an incoming beat and the beat is weaker beat. Hence, weaker and stronger beats arise alternatively creating variabilities. However, this mechanism does not explain the beat-to-beat variability, which in the model, involves inactivation of the Na^+^ channel. This phenomenon has been observed in previous simulation and experimental studies [77,78].

The loss-of-function mutation in RyR2 significantly diminishes its sensitivity towards the luminal Ca^2+^ and that causes the SR Ca^2+^ overload resulting in idiopathic behavior. But not enough explorations have been performed in this field, and more questions need to be answered. It is known that many instabilities carried out in the myocytes are by aberrant SR Ca^2+^ release, and certainly, there is a larger consequence of having the bulkier SR. In addition to RYR2-A4860, Roston et al. [74] reported another loss-of-function mutation, RyR2-I4855M, responsible for left ventricular non-compaction CPVT. A variation exists in types of RyR2 mutations, and there are also more CPVT variants; more work in this field will shed light on them.

There are many disagreements about loss-of-function hypotheses. Priori and Napolitano [79] stated that the proposed mechanism of the loss-of-function mutation [72] shifts away from the ground reality that the majority of the EADs are found in the setting of a low heart rate, and getting them the way they presented in their canine experiment is very unlikely. We also saw that the luminal dependency is the major modulator of RyR2 sensitivity, and any downturn in it affects the whole Ca^2+^ dynamics of the heart causing cardiac abnormality, like the way an increase in the luminal dependency affects the stability. As they also mentioned, the loss-of-function hypothesis is a provocative hypothesis and blame its divergence from the common knowledge of EADs, but we believe more study is necessary before concluding anything here.

### 4.5. Mechanism of EADs

Previous studies have explored the mechanisms of EADs. The observations have shown that during an EAD in heart failure, there is an increase in Ca^2+^ sparks, which can increase NCX [53,80]. Furthermore, these studies found that the rise in voltage is preceded by increases in both the L-type Ca^2+^ current and the NCX current, but they could not differentiate. EADs typically require prolongation of the AP to occur [81]. The simulations in this study with CPVT mutants show that although in a rapid pacing simulation with a gain-of-function mutant, there is more I_NCX_ than in a slow pacing simulation, with a loss-of-function mutant, there are only EADs in the latter. This and other evidence in the study (Table 2) suggest that in this case, the L-type Ca^2+^ channel is crucial for the initiation of the EAD. For the initiation mediated by the L-type current, the AP needs to be prolonged sufficiently to allow its reactivation during the plateau. The L-type current activation can trigger more Ca^2+^ sparks that can then activate NCX. In later EADs, the loop of LCC → RyR2 → NCX → LCC, etc., can occur prolonging the EAD with multiple humps. The contrast between the simulations in Figure 6 and Figure 9 also demonstrates this. The L-type Ca^2+^ current in the plateau where EADs initiate is about −4 µA/cm^2^. This is more than 2-fold higher than the depolarizing current generated by the NCX. Hence, its role is greater. That being said, a reduction in NCX, as shown in Figure 6, can reduce the EADs by reducing the plateau duration.

### 4.6. Guinea Pig Excitation-Contraction Coupling Modeling Milestones

Models of EC coupling in ventricular myocytes have developed following a similar pattern: (1) common pool deterministic models, (2) simplified local control models, and (3) detailed local control models. Models for the Guinea pig ventricular myocyte have followed this pattern. The Guinea pig is an important experimental model because it displays a long AP with a plateau more similar to the human, unlike the mouse and rat. Notably, Jafri and colleagues have already developed models for human and rat [53,77,82]. The first computation of the model of the Guinea pig ventricular myocyte was developed by Luo and Rudy [31,32]. This model was an advance on previous models of ventricular myocytes because it constrained the current of the sarcolemmal ionic channels with experimental data. However, it has a phenomenological model of Ca^2+^ release and was unable to produce the graded release due to its formulation as a common-pool model (one compartment with the L-type Ca^2+^ currents and the RyRs would interact [83,84]). Jafri and co-workers improved upon the Luo–Rudy model by incorporating a dyadic subspace compartment with biophysically detailed representations of the L-type Ca^2+^ channels and RyRs [29]. Capturing realistic biophysics enabled this model to reproduce interval force relations (the dependence of AP and Ca^2+^ transient amplitude and duration changes at different pacing rates) as an emergent property [29,85]. The termination of RyR Ca^2+^ depended upon depletion of the junctional SR. A decade later, Pasek and colleagues developed a Guinea pig ventricular myocyte model to study how ionic concentrations changed inside the t-tubular system and how this affected interval force relations [86]. Gauthier and colleagues incorporated a model that introduced simplified independent dyadic space compartments with a single RyR and a single L-type Ca^2+^ channel that produced graded release and restitution dynamics [87]. In this model, release termination depended upon strong inactivation of the RyR, which has not been experimentally verified. Simplification was necessary because of the computational complexity of solving a realistic stochastic model. Fortunately, more recently, with the advent of GPUs and faster CPUs, as well as advanced Monte Carlo algorithms, realistic detailed stochastic models have become possible. The current model presented in this manuscript is an example of a realistic detailed stochastic model of excitation–contraction coupling that improves upon the previous work [67,68].

### 4.7. Clinical Applicability of Study Findings

There are several insights gained from this computational study. The first is that simple SR Ca^2+^ in itself is not sufficient to trigger an arrhythmia. There needs to be an increase in RyR opening though Ca^2+^-induced Ca^2+^ release. Spontaneous Ca^2+^ release in the form of Ca^2+^ sparks can trigger an increased NCX current, which can help to maintain the AP plateau. However, reactivation of the L-type currents, which is essential for initiating EADs, triggers more RyR opening to start the cycle. Clinically, this cycle needs to be broken by reducing the L-type Ca^2+^ current using a Ca^2+^ blocker, as is done in the clinic for CPVT in combination with β-blockers. The β-blockers are important to prevent β-adrenergic stimulation, which the model demonstrates can lead to the conditions for arrhythmia. An alternative would be to reduce NCX; however, this could be problematic given that it could lead to Ca^2+^ overload. These results must be taken in context as this is a Guinea pig ventricular myocyte model and not a human ventricular myocyte model. Moreover, because these are model predictions, validation in experimental and clinical studies is required.

## 5. Conclusions

This quantitative model and analysis of variations caused by mutations studied the role of Ca^2+^ sparks in the subspace, β-adrenergic stimulation in action potentials (APs), Ca^2+^-related currents, transients, and intracellular Ca^2+^ storage. The study found the following:

- Reactivation of the L-type current was necessary for the initiation of the EAD. This would trigger additional Ca^2+^ sparks, which would in turn activate NCX extending the plateau and causing more L-type channel activation continuing the loop.
- AP variation and EADs are the underlying mechanisms to generate arrhythmia when the RyR2 mutant myocyte undergoes adrenergic stimulation either by exercise or stress or catecholamine perfusion during the condition of CPVT1.
- Fluctuation in intracellular Ca^2+^ dynamics due to alternation in SR Ca^2+^ transients, a diastolic interval, and diastolic Ca^2+^ load generate APD variations in the cardiac mutant myocytes. Similarly, the non-recovery of Na^+^ channels from the previous inactivation produces an alternate Na^+^ current (I_Na_); the alternate I_Na_ is responsible for producing an AP amplitude variability.
- The increased heart rate required to generate arrhythmia, but rapid pacing itself, is not enough to generate arrhythmia.
- I_ncx_ increases the APD duration in the variabilities based on its electrogenic property, and the frequency of EADs can be minimized by blocking this channel. It has a positive feedback mechanism with I_LCC_.
- SR leak is highly dependent on the diastolic SR Ca^2+^ volume. But an increase in the release via RyR2 is not going to help it, and so leaked Ca^2+^ is not enough to generate DADs.
- The SR Ca^2+^ available for the current beat is equally as important as the overall SR load to bring Ca^2+^ instability in the cardiac myocytes.

## Figures and Tables

**Table 1:** Modulation parameters in the for RyR2 mutation simulations.

Simulation Types	Half-Maximal Point (K^−^)	Hyperactivity (K^+^)	Luminal Dependency (K_JSR0_)	Allosteric Coupling $\left(a_{*}\right)$
Gain-of-function	Decrease	Increase	No change	Decrease
Loss-of-function	No change	No change	Decrease	No change
Binding Protein	No Change	Increase	No change	Decrease
SOICR	No change	No change	Increase	No change

**Table 2:** Depolarization of I_LCC_, I_ncx_, and AP and opening of RyR2 in EADs.

EAD Number	Time of Initiation of Each Component (s)			Differences in Initation Times (s)	
EADs	I_LCC_	RyR2	I_ncx_	RyR2-I_LCC_	I_ncx_-RyR2
EAD1	12.12	12.14	12.14	0.018	0.009
EAD2	12.22	12.22	12.23	0.009	0.001
EAD3	12.33	12.34	12.35	0.014	0.003
EAD4	16.13	16.16	16.19	0.029	0.024
EAD5	16.22	16.24	16.25	0.021	0.008
EAD6	16.40	16.42	16.44	0.027	0.019
EAD7	17.17	17.18	17.19	0.010	0.017
EAD8	17.25	17.29	17.30	0.034	0.016
EAD9	18.17	18.19	18.20	0.024	0.010
EAD10	19.16	19.18	19.19	0.018	0.012
EAD11	19.32	19.35	19.36	0.024	0.011
			Mean	0.021	0.012
		Standard Deviation		0.008	0.007

**Figure A1:**
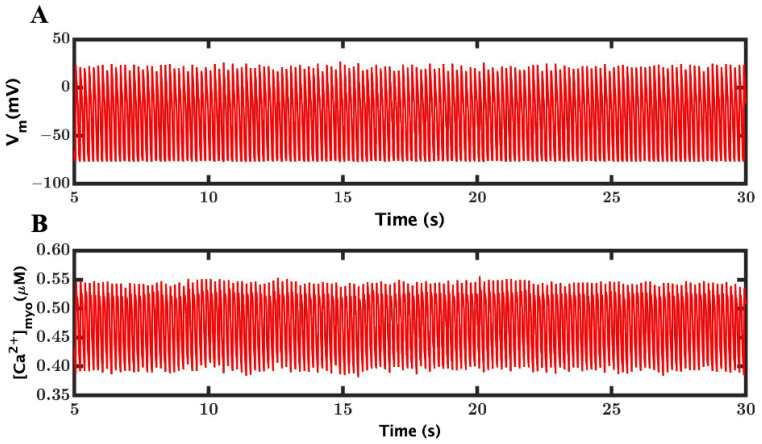
A 30 s simulation of the gain-of-function mutant shown in Figure 9, Figure 10 and Figure 11, showing increased variability. (**A**) Action potential. (**B**) Myoplasmic [Ca^2+^] transient.

**Figure A2:**
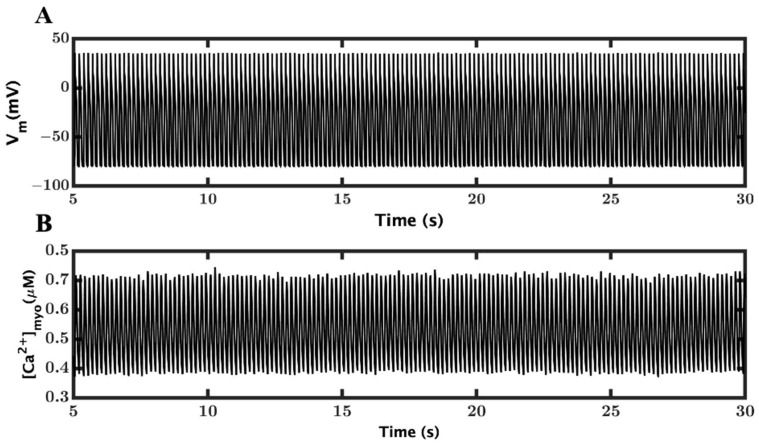
A 30 s simulation of the wild-type myocyte variability shown in Figure 9, Figure 10 and Figure 11. (**A**) Action potential. (**B**) Myoplasmic [Ca^2+^] transient.

## Data Availability

Data are contained within the article.
